# Polydomain Liquid Crystal Elastomers with Mechanically Switchable Opacity for Thermal Shielding

**DOI:** 10.1021/acspolymersau.6c00032

**Published:** 2026-04-17

**Authors:** Marco Turriani, Andrea Lanfranchi, Diederik S. Wiersma, Camilla Parmeggiani, Paola Lova, Daniele Martella

**Affiliations:** † Dipartimento di Fisica e Astronomia, 9300 University of Florence, Via Sansone 1, 50019 Sesto Fiorentino (FI), Italy; ‡ LENS (European Laboratory for Non-Linear Spectroscopy), Via Nello Carrara 1, 50019 Sesto Fiorentino (FI), Italy; § Dipartimento di Chimica e Chimica Industriale, University of Genoa, Via Dodecaneso 31, 16146 Genoa, Italy; ∥ Dipartimento di Chimica “Ugo Schiff”, University of Florence, Via della Lastruccia 13, 50019 Sesto Fiorentino (FI), Italy

**Keywords:** liquid crystal elastomers, smart screens, responsive optical materials, thermal shielding, polydomain liquid crystals

## Abstract

Smart screens with adjustable optical properties represent an emerging technology for functional light management. Applications range from temperature control, both for indoor and outdoor environments, to innovative lighting solutions. To date, most of the materials employed in the field require a continuous energy supply to maintain the desired optical properties, while others directly responding to the environment are difficult to control and hardly tunable. In this work, we demonstrate an alternative solution based on polydomain liquid crystal elastomers (LCEs) with reversible mechanical modulation of their opacity. Our mechano-responsive LCEs provide optical scattering properties that are easily controllable and self-maintained without energy-consumption. The materials are prepared by a low-cost, easily scalable one-step procedure with commercially available monomers. The synthesized LCEs present a colorless, opaque appearance that can be gradually and reversibly transformed into a transparent monodomain material by applying a tensile stress. Beside shading properties, the mechano-responsive LCEs allow a strong thermal shielding effect diminishing the temperature rise up to 10 °C upon irradiation with simulated sunlight when employing the opaque state. This work establishes an important step for new smart screen technologies not needing complex fabrication techniques nor a continuous power supply for their dynamic operation.

## Graphical abstract

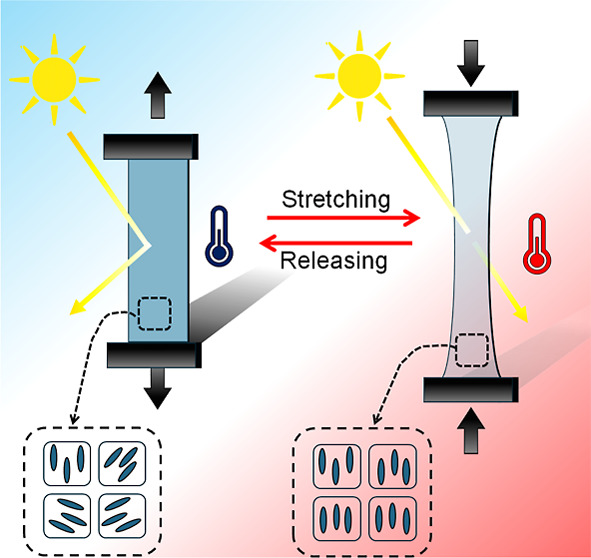

## 1 Introduction

Smart materials with adjustable optical properties represent a versatile solution for emerging light-management applications. Both smart screens and smart windows are increasingly in demand to provide customized indoor comfort and improve energy efficiency. Beyond these uses, these materials are also gaining significant attention for physical information encoding and for advanced camouflage and anticounterfeiting systems.^1–5^ Indeed, smart screens can be used to regulate artificial interior lighting or to create spaces that can be dynamically partitioned, with the possibility to switch between a privacy mode (opaque screen) and a public one (transparent). Windows, in contrast, serve as an essential interface between indoor and outdoor environments, providing natural illumination as well as allowing solar thermal radiation to enter.^6^ Notably, windows are recognized as the least energy-efficient component in buildings, accounting for approximately 60% of overall energy loss.^7^ Smart windows with tunable regulation of sunlight transmittance can improve energy performances and lead to substantial savings.^8,9^ In cold conditions, they show high transmission in both the visible (380–780 nm) and near-infrared (780–2500 nm) spectral ranges to promote indoor heat gain. On the other hand, during warmer seasons, they should reduce their transparency in the infrared part of the spectrum.^6,10,11^

Development of all these applications requires responsive materials capable of undergoing reversible changes of optical appearance, including variations of color and opacity (light scattering).^12^ Based on their operating principle, these materials have been traditionally grouped into three main categories: electro-, photo-, and thermoresponsive. Between them, electroresponsive materials are the most extensively studied and rely on the reversible change in absorbance or scattering upon applying voltages. They can be realized exploiting redox reactions in metal oxides^13^ or inducing order/disorder states in liquid crystals or microparticles dispersed in polymeric matrices.^9^ Electrochromic windows offer excellent controllability and fast switching time (on the order of milliseconds). However, they require a continuous power supply to maintain the “on state” (e.g., colored mode in metal oxide-based and transparent in liquid crystals). Photo- and thermoresponsive materials have been proposed to lower energy consumption by reacting to environmental stimuli.^14,15^ These systems offer many advantages, such as ease of installation and the absence of external power requirements. However, because they are passive and lack active controllability, they may become inefficient or even counterproductive under certain conditions, especially for thermal management.

To address the limitations associated with energy consumption and the lack of controllability, a fourth category of smart materials has recently emerged: the mechano-responsive one, which modifies their transmittance in response to mechanical stimuli. Current examples include micro- or nanostructured materials varying optical properties by strain application.^16^ Previous studies describe systems featuring surface wrinkles or cracks that switch reversibly from opaque to transparent upon stretching, or patterned surfaces with periodic nanoscale or microscale arrays of nanocones, nanopillars, nanoholes, or nanospheres.^17^

Although state-of-the-art mechano-responsive materials represent an optimal solution in terms of both energy consumption and controllability, micro- and nanopatterning requires specialized fabrication techniques, which limits the scalability of smart screens and windows. In this context, (unpatterned) polymeric films based on liquid crystal elastomers (LCEs) with adjustable transmission can represent a versatile solution. LCEs are smart materials extensively studied for their fast and reversible shape change in response to external stimuli such as temperature, light, or chemicals.^18–23^ The precise control of the liquid crystal alignment determines both the direction and magnitude of actuation,^24–26^ enabling the development of microactuators for soft robotics^27–29^ or photonic devices.^30,31^ Interestingly, when LCE fabrication is further simplified and no external field is applied during the network formation (to impose a specific molecular orientation), the material develops a polydomain texture that is macroscopically isotropic yet exhibits a high degree of local nematic order on the micrometric or nanometric scale. Current applications of polydomain LCEs include high damping elements,^32^ tunable adhesives,^33^ and supports for stretchable electronics.^34^ In these materials, mesogens self-assemble into multiple birefringent domains with sizes comparable to the wavelength of visible light, each featuring randomly oriented nematic directors (optical axes). This morphology produces strong light scattering, which is responsible for their high opacity. Upon heating, weakly cross-linked LCEs undergo a reversible transition from the nematic to isotropic phase, where birefringence disappears and the material becomes optically transparent, in analogy to low-molecular-weight LC behavior. In other cases (and in dependence of the cross-linking density), transparency under heating is more properly described by a molecular rearrangement of the domains, which lower the light scattering.

A second tuning mechanism, very appealing for the realization of smart screens and windows, is given by their mechanoresponsive behavior, as sketched in Figure 1. In detail, applying a tensile stress gradually rotates and aligns the nematic domains along the shear direction, transforming the material from an opaque polydomain state to a transparent monodomain one. This optical property was first documented by Finkelmann and co-workers with subsequent studies mainly focused on the modeling of LC domain reorganization during the polydomain–monodomain transition.^35–38^ Only more recently, polydomain LCEs have been proposed as optical materials with adjustable transmission allowing, for example, physical information encryption^39^ or the realization of tunable masks for biomimetic camouflage.^40^ However, a comprehensive quantitative evaluation of the optical and thermal shielding properties of these materials, essential for their use as smart screens or thermal shielding devices, is still missing.

**Figure 1:**
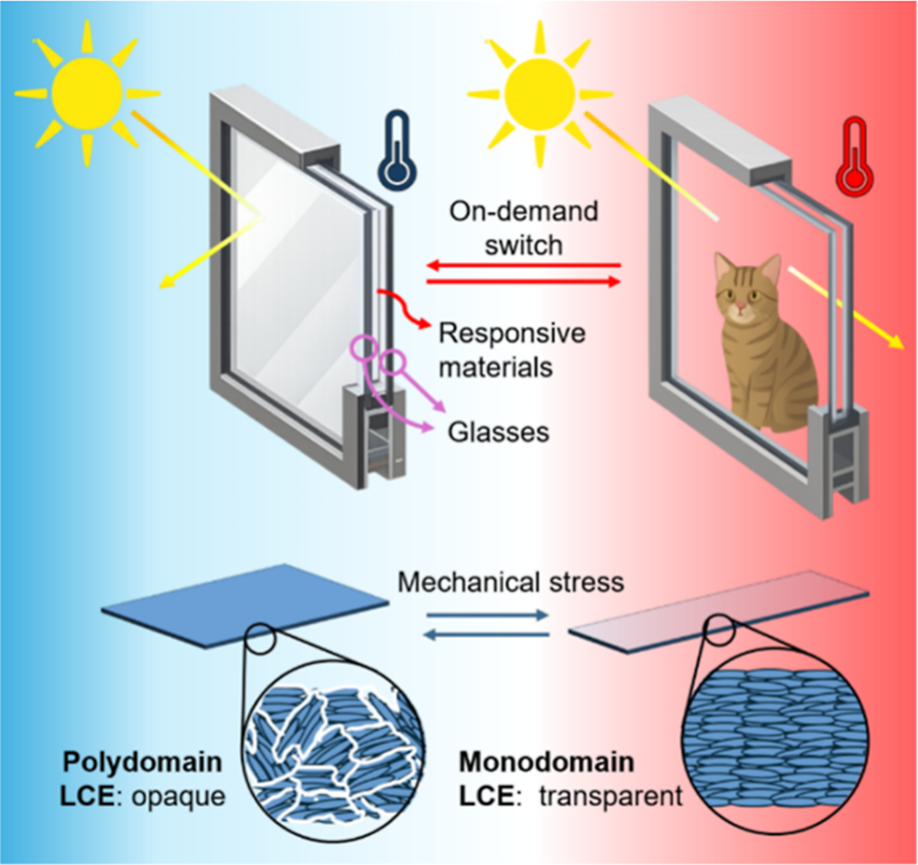
Scheme of a smart screen and working principle of LCEs with mechanically switchable transparency.

The strain-induced transparency has been described by some authors as “transparency change mechano-chromism”,^41–43^ while others distinguish it from chromatic mechano-chromism which involves wavelength shifts.^44,45^ To avoid ambiguity, we refer to this behavior as “mechanically-switchable transparency”.

This work demonstrates the synthesis and characterization of a series of polydomain LCEs promising for both interior smart screens and exterior smart windows. The selected synthetic strategy relies on a simple thermal curing of a monomeric mixture without requiring any control over molecular alignment, lithographic patterning, or additional pre- or post-treatments. The resulting polydomain LCEs exhibit thermoresponsive transparency, with a progressive increase in the optical clearing point as the cross-linking density rises, as well as a strong thermal-shielding effect in the polydomain phase. This aspect has been studied under dynamic strain conditions, demonstrating that LCEs undergo fully reversible mechanically switchable transparency, achieved by optimizing the initial stress applied to the material. Of note, once the stress-induced optical state is set, the LCEs remain stable until the mechanical stimulus is modified, removing the need for a continuous power supply to maintain the optical tuning, an important advantage compared to previously reported LC-based smart windows or screens.

## 2 Experimental Section

### 2.1 Materials

The liquid crystalline monomer 2-methyl-1,4-phenylene *bis*(4-(3-(acryloyloxy)­propoxy)­benzoate) (RM257) was purchased from Synthon Chemicals. 2,2′-(Ethylenedioxy)­diethanethiol (EDDET), pentaerythritol terakis­(3-mercaptopropionate) (PETMP), triethylamine (TEA), poly­(vinyl alcohol) (PVA), and dichloromethane were purchased from Merck.

### 2.2 Film Preparation

All of the LCN films were prepared following the same procedure. Here, we reported the synthesis of LCN30 as an example. 46 mg of PETMP (0.094 mmol) and 71 μL of EDDET (0.44 mmol) were mixed with 7 μL of triethylamine (0.05 mmol, 1% weight) in a vial. 369 mg of RM257 (0.63 mmol) was dissolved in 1 mL of dichloromethane, filtered over cotton, and poured into a vial. The homogeneous solution was cast over a microscope glass slide (76 × 26 mm^2^) previously covered with a 5% PVA solution in water. The cast glasses were left at RT for 2 h and then put in an oven at 90 °C for 15 h. Films were detached from the glass support with a blade after immersion in water for 1 h. The quantities of reagents used for the preparation of the other materials are reported in Table 1.

**Table 1:** LCEs Composition and Thermomechanical Characterization

	monomer mixture composition				DMA		
	RM257 (eq)	EDDET (eq)	PETMP (eq)	TEA (% weight)	*T*_g_ (°C)	*G*′_glassy_ ^a^ (Pa)	*G*′_rubbery_ ^b^ (Pa)
**LCE20**	1	0.8	0.2	1	0	3.4 × 10^6^	3.4 × 10^4^
**LCE30**	1	0.7	0.3	1	0	2.0 × 10^6^	2.4 × 10^4^
**LCE50**	1	0.5	0.5	1	5	1.4 × 10^6^	9.1 × 10^3^

a Shear storage modulus at −15 °C.

b Shear storage modulus at 95 °C.

### 2.3 Characterization

The polymerization was monitored by attenuated total reflectance infrared spectroscopy (Shimadzu IRAffinity-1S). The gel fraction was measured through a gravimetric method in which the materials were weighted before and after being immersed in dichloromethane for 72 h. Storage modulus and glass transition temperatures were measured through a dynamic mechanical analysis (DMA) PerkinElmer DMA 8000. The measures were performed in shear geometry on specimens of 5 × 5 mm^2^, at frequency 1 Hz, and a strain of 0.2%. The temperature was increased from −20 to 100 °C with a heating rate of 2 °C/min. The thermochromic behavior was evaluated using a custom setup consisting of a UV/vis lamp (Ocean Insight/Optics DH-2000-BAL), a spectrophotometer (Ocean Insight/Optics RedTide USB650), and a Linkam PE120 hot stage. The measurements were recorded in heating and cooling at 10 °C/min with an isothermal of 1 min every 10 °C.

### 2.4 Simultaneous Measurement of Switchable Transparency and Thermal Shielding

The opto-thermomechanical characterization was performed using a custom-built optical setup integrated into a tensile machine (Instron 5565), as shown in Figure 6. Samples were first cut to 1 cm-wide and 6 cm-long stripes and then held by the clamps of the tensile machine to ensure a useful length of 4 cm. Light from the source (Newport Oriel LCS-100 Solar simulator) impinges on the sample from the front and, through it, on the bottom side of the integrating sphere placed behind (Avasphere 5 cm, 0.5 cm port width). The distance between the integrating sphere and the solar simulator is 17.8 cm, equivalent to 1 Sun of power as per the instrument’s certification. The integrating sphere collects the light transmitted through the sample and sends it to a UV–vis spectrometer (AvaSpec-ULS2048CL-EVO, spectral range 250–1100 nm, resolution 1.3 nm) via optical fibers. Two thermistors (Thorlabs TSP01B, range −20/120 °C, accuracy 0.5 °C) are taped each to a 1 cm^2^ expanded polystyrene block fixed to the bottom of the integrating sphere. One thermistor is taped below the sample port, screened by the LCE sample, the other is directly exposed to irradiation from the solar simulator. This way, as the tensile machine performs stress–strain measurements on the samples, their transmittance can be monitored by the integrating sphere/spectrometer system, and the thermal shielding effect can be evaluated monitoring the temperature of the two thermistors, with the unscreened one acting as a reference. We expect thermal conductivity and emissivity of the sample to have a negligible effect on the final temperature reached by the thermistor. This is due to the sample–thermistor distance, the high power of the lamp in the UV/vis/NIR range, and the low temperature reached by the sample itself, reducing its own emission. The thermal pictures and video were recorded using a FLIR T560 thermal camera (Teledyne FLIR LLC, USA). The camera was equipped with a Germanium lens and a 24° viewing angle objective (resolution 640 × 580 pixel, measurement range of 0–650 °C, accuracy of ±2 °C or ±2% of reading, sensitivity of <40  mK at 30 °C). The thermistors were removed, while three stripes of black tape were placed respectively behind the sample (on the upper and lower sides of the integrating sphere’s port) and directly exposed to the solar simulator (on the left side of the sample port).

## 3 Results and Discussion

### 3.1 Material Preparation and Characterization

The preparation of polydomain LCEs was achieved through a one-step strategy involving a base-catalyzed thiol–Michael copolymerization, as shown in Figure 2a,b. Thin films were obtained through the casting of a monomer solution containing a diacrylate liquid crystal (RM257), a dithiol (EDDET, used as a chain extender), a tetrathiol (PETMP, used as a cross-linker), and triethylamine as the basic catalyst. During the thermal curing, the thiol groups (red parts in Figure 1a) give addiction on the β-carbon of the acrylate groups (green parts in Figure 1a) to form a C–S bond resulting in the formation of linear chain oligomers (EDDET can bond 2 RM257 molecules) and in their cross-linking (4 bonds created by PETMP). To study the effect of cross-linking density on the mechanical and optical properties, we prepared three formulations containing different equivalents of each component. A complete list of the monomer mixtures is reported in Table 1. The ratio between acrylate and thiol groups was maintained constantly 1:1 in all the formulations. while the ratio between the equivalents of the chain extender and the cross-linker were respectively 80:20, 70:30, and 50:50 for the materials called LCE20, LCE30, and LCE50. Indeed, increasing the tetrathiol concentration allows an increase in the cross-linking density with a consequent impact on the mechanical properties of the resulting polymer. The monomer solutions were cast over microscope glass slides and left at room temperature, allowing the monomers to react. After 2 h, we observed the formation of optically transparent films that were subsequently heated to 95 °C for 15 h to increase the conversion and complete the solvent evaporation. At the end of the procedure, LCE20 and LCE30 films appeared homogeneously white at room temperature; conversely the most cross-linked material (LCE50) was semitransparent (Figure 2c). The different material appearance can be explained by the different domain sizes of the liquid crystal structure, as reported by polarized optical microscopy (POM) observation in Figure S1.

**Figure 2:**
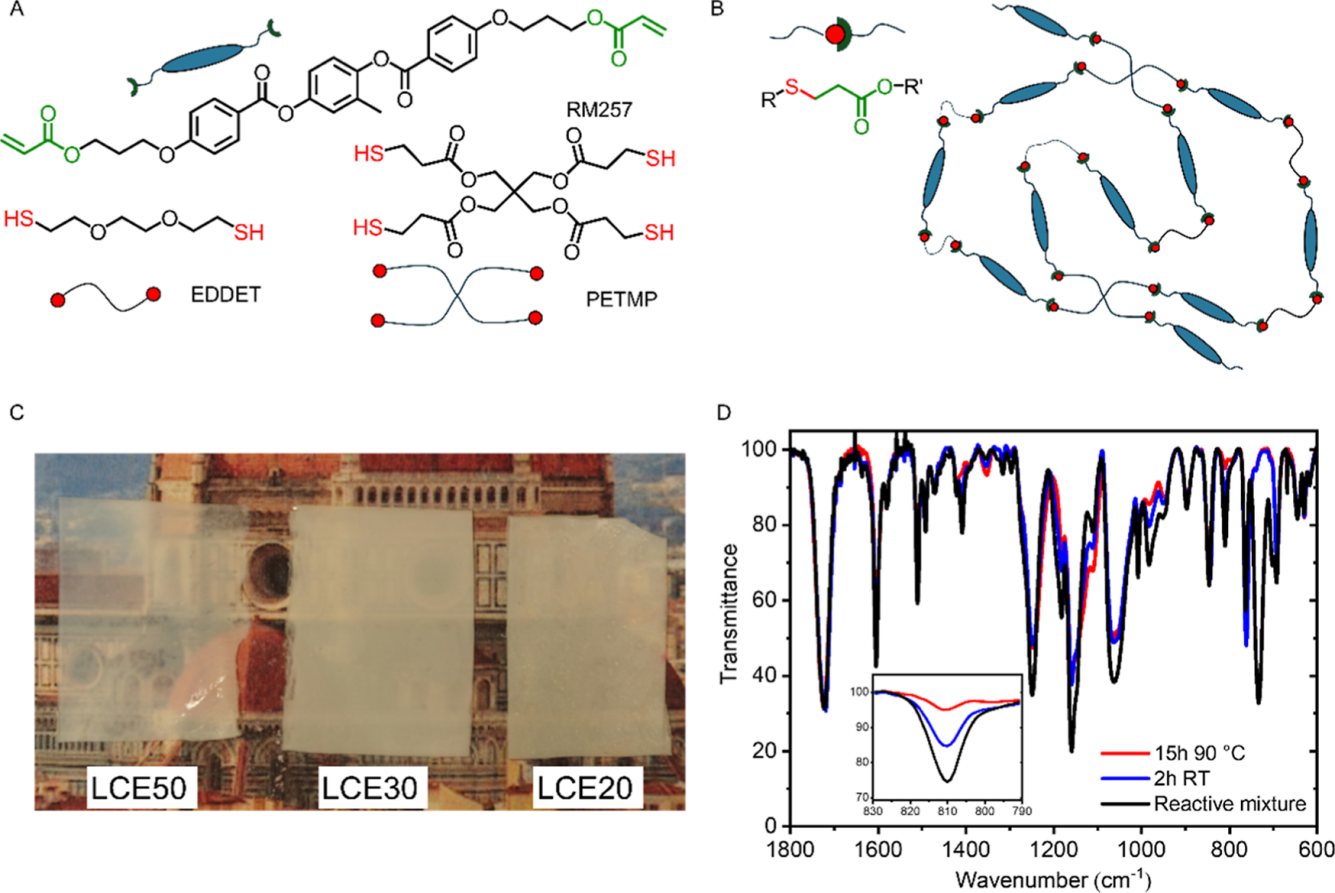
Preparation and characterization of polydomain LCEs. (A) Structures of the monomers involved in the polymerization. (B) Graphical representation of the LCE structure. (C) Photographs of LCE50, LCE30, and LCE20. (D) ATR-IR spectra of the reactive mixture of LCE30 (black), after 2 h at room temperature (blue) and after 15 h at 90 °C.

Successful polymerization was confirmed by attenuated total reflectance (ATR-IR) spectroscopy, monitoring the disappearance of characteristic acrylate bands (1410 cm^–1^, C=C stretching, and 811 cm^–1^, =CH_2_ bending, Figure 2d). As an example, for LCE30, the conversion of acrylate groups calculated from ATR spectra, after the first 2 h at room temperature, was 42% and increased up to 86% after the thermal treatment. However, the gel fraction calculated from gravimetric methods on the same material was about 98% indicating an almost complete incorporation of the monomer inside the network.

Differential scanning calorimetry (DSC) of the final materials allowed to determine the glass transition temperature with no other transition peaks clearly visible (see Figure S2), as expected and extensively reported in previous studies for cross-linked LCEs.^46,47^ The thermomechanical behavior of the LCEs was then investigated through DMA, and data are reported in Table 1. Representative curves of the shear storage modulus (*G*′) and tan­(δ) of all samples are also reported in Figure 3. At low temperatures, the materials exhibited a storage modulus in the order of MPa (10^6^ Pa), while after the jump in correspondence with the glass transition, the G′ curves showed a rubbery plateau and the modulus varied from 9.1 × 10^3^ Pa for LCE20 to 3.4 × 10^4^ Pa for LCE50 in accordance with the higher cross-linking density. In correspondence with the jump in G′ curves, tan­(δ) curves showed a maximum, associated with the glass transition temperature (*T*_g_) that varied from 0 °C for LCE20 to 5 °C for LCE50. Above the *T*_g_, tan­(δ) curves showed a second peak (very broad in LCN20 curve), already observed for similar LCEs and associated with the rubber-nematic state in which domain rotation and viscous deformation of non-mesogenic segments efficiently dissipated mechanical energy.^48^

**Figure 3:**
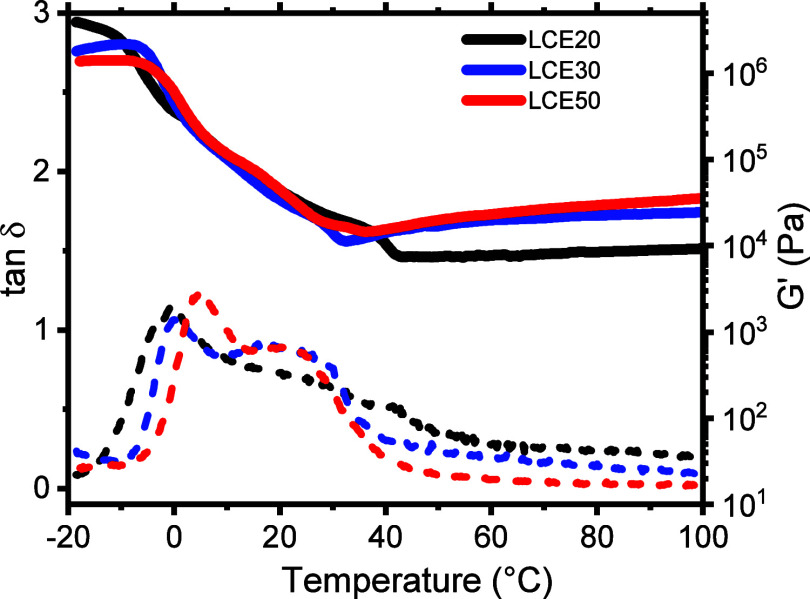
Dynamic Mechanical Analysis. Shear storage modulus and tan­(δ) curves for the synthesized LCEs.

### 3.2 Thermoresponsive Behavior of Polydomain LCEs

A thorough investigation of thermo-switchable transparency of LCEs is essential for their use as smart windows in conditions where temperatures may exceed 40 °C. In particular, it is necessary to define the temperature operability window in which the optical switch can be triggered exclusively by mechanical means. This is necessary to avoid external environmental factors (e.g., hot days during summer) competing with the regulation of light transmission by the operator. Indeed, upon heating, a decrease in the nematic order of the LCEs can induce a gradual reduction of the birefringence and of the scattering due to the polydomain structure. Heating up the materials, they become gradually more transparent until a characteristic threshold temperature, where they become completely clear (Figure 4a). Observations at the polarized optical microscope (POM) during the heating cycle revealed a incomplete loss of the material birefringence (Figure S3), suggesting that the molecular rearrangement of the polydomain structure is the driven mechanism for material transparency. Indeed, POM observations on LCE50 during a heating and cooling cycle were very useful to demonstrate how the domain size increases with temperature and returns to the original structure when cooled at room temperature (Figure S4).

**Figure 4:**
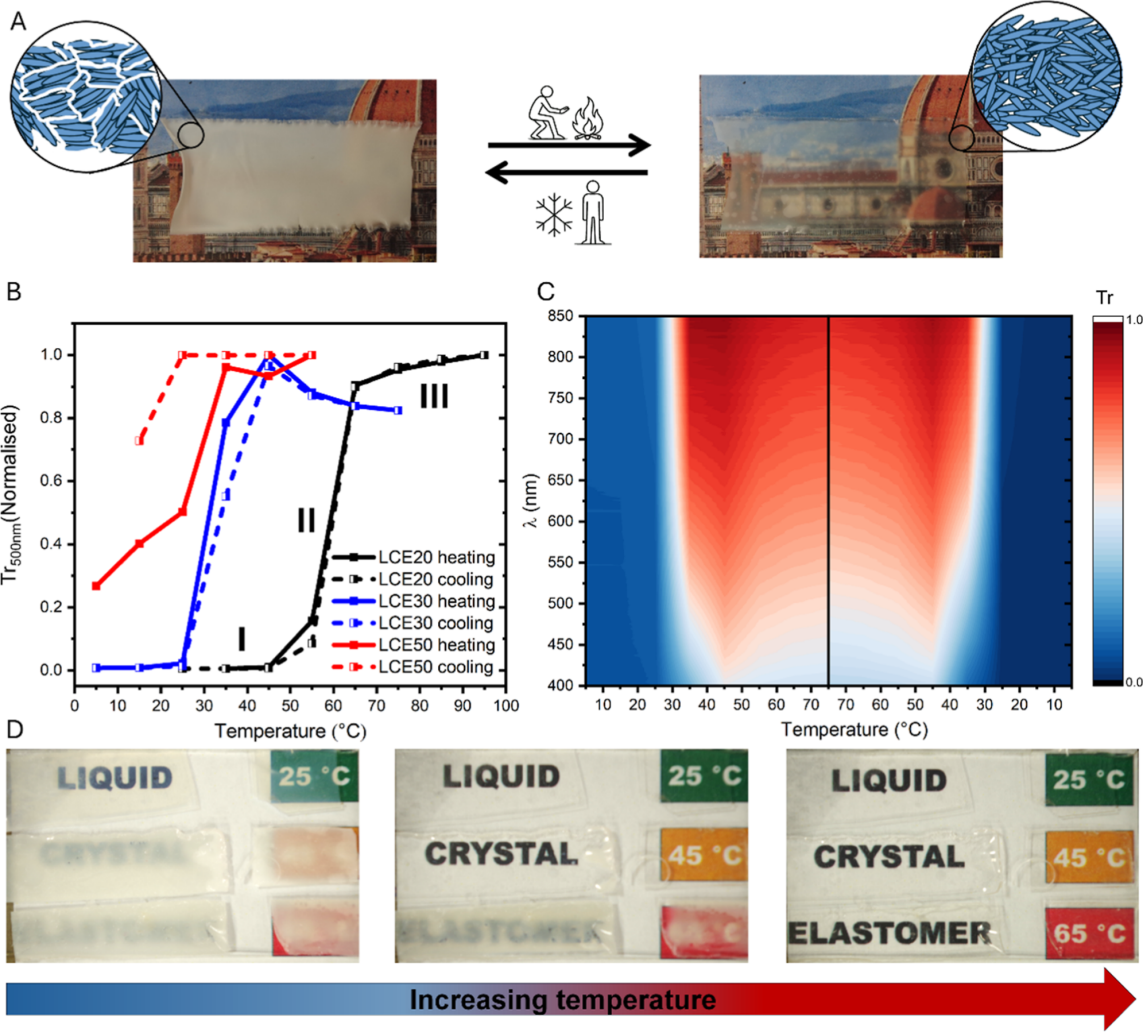
Thermoresponsive behavior of LCEs. (A) Picture of a polydomain LCE during a heating–cooling cycle. (B) Transmittance (Tr) of LCEs at 500 nm normalized by the maximum value over different temperatures. (C) Transmittance spectra over different temperatures for LCE30. The data report the evolution of the spectra during a whole heating and cooling cycle. (D) A proof-of-concept for physical data encryption inside polydomain LCEs: photos of LCE50, LCE30, and LCE20 (from the top to the bottom) at 25, 45, and 65 °C (from left to right).

A more quantitative comparison of the thermoresponsive optical behavior is reported in Figure 4b, showing the transmittance (Tr) of ballistic light at 500 nm over different temperatures. As an example, the complete thermal characterization of LCE30 is reported in Figure 4c. The transmittance–temperature traces of LCE20 and LCE30 (Figure 4b) follow a sigmoidal evolution: at lower temperatures (below 25 °C for LCE30 and below 45 °C for LCE20, region I), the samples diffuse efficiently the light, resulting in low transmittance (Tr < 0.05); as the temperature increases, transmittance begins to rise in a pseudo-linear way over a temperature interval of approximately 20 °C (region II), reaching a maximum where a second plateau appears (materials were clear and highly transparent, region III). Upon cooling, the phenomenon was fully reversible, with complete recovery of the sample opacity and without evident hysteresis phenomena (dashed lines in Figure 4b) in the transmittance–temperature traces. On the other hand, LCE50 follows a similar trend, but due to instrumental limitations, the low-temperature plateau could not be reached and should be present below 0 °C. Additionally, the cooling curve exhibits a slight hysteresis that could be determined not only by a difficult rearrangement of the more cross-linked structure but also by intrinsic limitation of our experimental setup.

The macroscopic “optical clearing point” (i.e., the temperature at which normalized transmittance reaches a value of 0.5) increases while cross-linking density decreases: from 25 °C for LCE50 to 35 °C for LCE30 and up to 60 °C for LCE20, demonstrating how varying the cross-linking density represents an easy and effective strategy to tune the thermo-switchable transparency of the LCEs. This behavior expands the interest of these materials also in the field of physical data encryption.^39,49,50^ Indeed, materials revealing specific information by observable property variation (e.g., color or shape) in response to specific external stimuli (the physical decryption key) are highly demanded. In this sense, our materials can hide basic information in the opaque state of the LCE and reveal them only at a specific temperature because of the thermoresponsive behavior just described. As proof of concept, Figure 4d shows an example of data encryption by combination of polydomain LCEs: at room temperature, only LCE50 was semitransparent, allowing the information underneath to be clearly red. Employing as physical decryption keys other two temperatures (one in between the different clearing points and another above them), the text behind the LCEs is gradually shown. Indeed, upon heating to 45 °C, LCE30 became transparent, revealing a second piece of information previously hidden behind it. Finally, upon heating to 65 °C, LCE20 also became transparent, and the complete message was revealed.

### 3.3 Mechanically Switchable Transparency and Thermal Shielding

The two materials with the lower cross-linking density (LCN20 and LCN30), having an opaque appearance at room temperature, can be modulated also in response to a uniaxial mechanical stress, showing a marked transition from the opaque to the transparent. This optical variation is due to the gradual rotation of the local director in the nematic domains (along the tensile stress direction) with the increasing mechanical stress until the formation of amonodomain sample.

A preliminary evaluation of the material behavior under mechanical stretching is shown in Figure 5 for LCE30. The film becomes highly transparent in the entire visible and near-infrared spectral range when stretched up to about 110% (state 1 to state 2), and upon stress release, it shows a partial shape recovery (up to strain, ε, = 50%) and a marked reduction in transmittance (state 2 to state 3). The two optical states at ε = 50% and ε = 110% are completely reversible, and it is possible to switch from one to the other simply by applying stress or releasing. Moreover, upon heating above the clearing point, the LCEs completely recover the initial shape and, after cooling, also the initial opacity (state 3 to state 1). Partial shape recovery in the nematic state of LCEs is a well-known and extensively studied phenomenon, attributed to the relatively long relaxation time associated with the rotation of liquid crystal microdomains.^51–53^ During POM observation, the stretched LCEs’ transmittance varies when rotated, showing maximum brightness when the stretching axis is at 45° with respect to cross polarizers (Figure S5). This behavior is typical of planar homogeneous monodomain LCEs and confirms the effective alignment of microdomains along the stretching direction.

**Figure 5:**
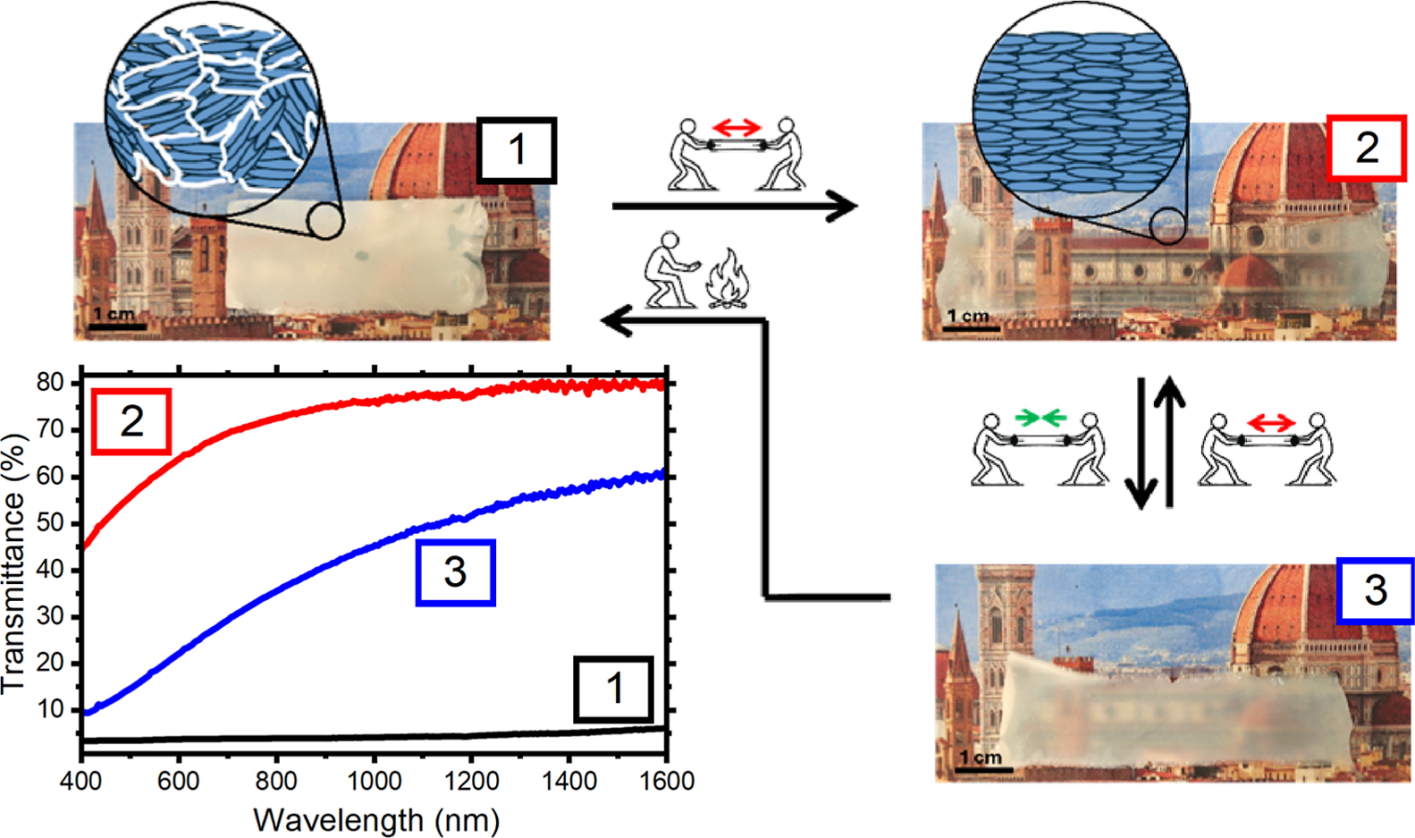
Mechanically switchable transparency. The LCE30 film was stretched up to 110% (state 1 → state 2), then the load was removed (state 2 → state 3), and the specimen partially recovered the initial shape and opacity. States 2 and 3 were fully reversible, and it was always possible switching between them loading and unloading the specimen. On the bottom in the left, the transmission spectra (400–1600 nm) of LCE30 corresponding to the three states (a–c) are reported.

This mechano-responsive behavior can be confirmed and quantified by performing both a single stress–strain test and several tensile cycles (elongation–relaxation) while continuously monitoring the sample transmittance using a spectrometer coupled with an integrating sphere. This test makes it possible to measure the temperature in the area shaded by the sample using a thermistor, thereby also quantifying the thermal shielding effect of the LCE. In this way, the reversibility of both thermal-shielding performance and optical variations can be directly monitored throughout the tensile test. A schematic of the setup used for these simultaneous measures is reported in Figure 6 and pictures in Figure S6.

**Figure 6:**
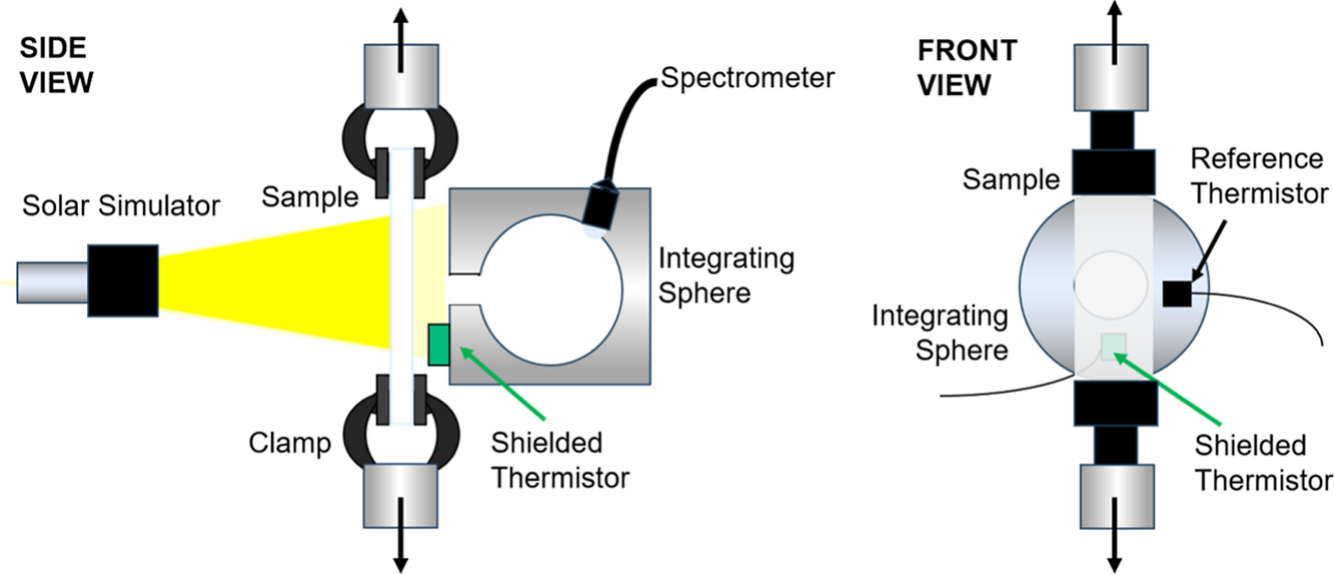
Schematics of the setup used to simultaneously study the mechanically switchable transmitted light and thermal shielding.

Samples (LCE20 and LCE30) were initially stretched up to 125% strain, and then the load was released to observe the elastic recovery. For both the samples, the stress–strain curves were qualitatively similar: after an initial short elastic region (ε = 0–6%), the curves displayed a plateau, followed by a second elastic region (Figure 7a,e). The plateau extended up to 81% strain for LCE20 and 68% for LCE30 and can be explained with the rotation of some of the microdomains less constrained by the polymeric network that align parallel to the tensile axis with the minimum energy consumption. This phenomenon is well-known for LCEs and represents the so-called soft elasticity plateau.^54^ After the plateau, a second elastic regime was observed where the modulus increased linearly with strain, reaching an applied stress of 0.17 MPa for LCE20 and approximately twice that value (0.33 MPa) for the more cross-linked LCE30 at ε = 125%. As expected, upon load removal, the more cross-linked sample, which also showed the narrower soft elasticity plateau, exhibited a greater elastic recovery (ε = 51% for LCE30 vs ε = 70% for LCE20). From this first observation, we identified the optimal strain region of our materials (corresponding to the material stretching from 75% to 125% of their length) for the construction of smart windows with reversible and controllable optical properties.

**Figure 7:**
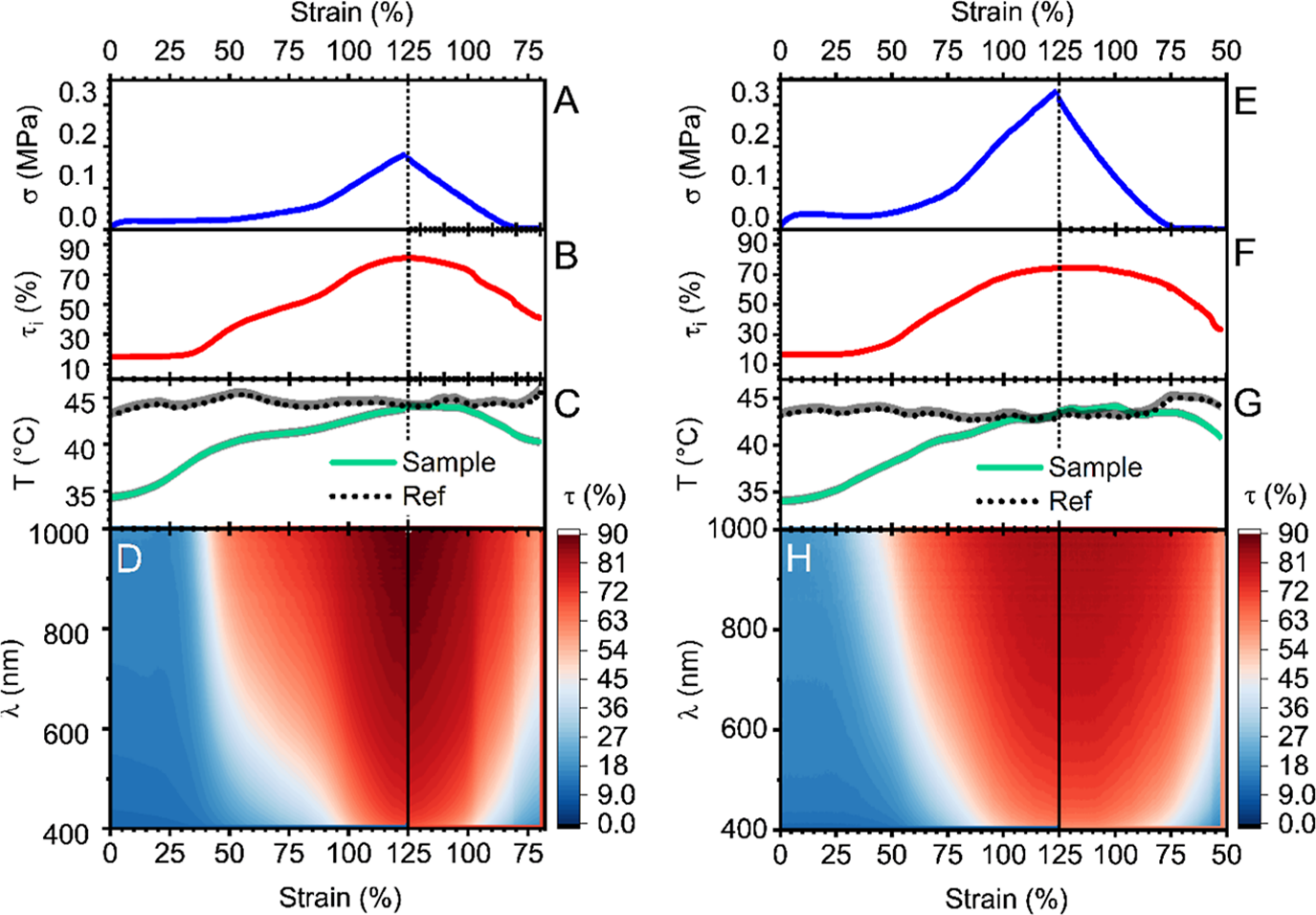
Relationship among strain, transmittance, and thermal shielding. (A, E) Stress–strain curve for LCE20 (left) and LCE30 (right) samples during pulling (first part) and retracting (second part) of the clamp position. (B, F) Integrated transmittance (in the range from 400 to 1000 nm) at different strain values for LCE20 (left) and LCE30 (right). (C, G) Temperature measured for the shielded thermistor (green curve) and for the reference thermistor (black dotted curve) at different LCE strain values. The shaded area yields the experimental error. (D, H) Transmittance spectra over different strain values for LCE20 (left) and LCE30 (right).

Figure 7b,f shows the integrated transmittance (τ_i_) of the sample collected during the tensile tests. τ_i_ is defined as the area under the transmittance spectra between 400 and 1000 nm. With increasing strain, the curves exhibited a sigmoidal trend: after an initial region (for ε = 0–37% in LCE20 and ε = 0–43% in LCE30), where the transmittance remained constant (τ_i_ ∼ 14%), it increases until reaching a plateau at ε ∼ 105% (τ_i_ = 80 for LCE20 and τ_i_ = 73 for LCE30). Interestingly, the change in transmittance did not occur in perfect correspondence with the strain region of the soft elasticity plateau: transmittance begins to increase approximately halfway through the plateau (even later in LCE30) and reaches its maximum in the elastic region. As already observed for the shape recovery, the more cross-linked LCE also showed a higher transmittance decrease (τ_i_ = 40 for LCE20 and τ_i_ = 32 for LCE30). Figure S8 in Supporting Information reports the optical contrast of the two materials.

The ability to shield light radiation in a controlled manner has immediate thermal repercussions on the area shielded by LCEs. Indeed, an object shielded by an LCE in opaque mode will have a lower temperature than the same object shielded by an LCE in transparent mode, as illustrated in the images obtained with a thermal camera during a stress–strain experiment in Figure S8 and Movie S1. A more quantitative evaluation of the LCE shielding capacity was obtained by comparing the temperature collected in real time during the tensile test by a thermistor screened by the sample and the temperature collected by an unshielded thermistor used as a reference (Figure 7c,g). At ε = 0, transmittance was minimal, and the temperature difference between the shielded thermistor and the reference (Δ*T*) was approximately −10 ± 1 °C for both samples. As the stretching increased, the temperature of the shielded thermistor increased, following a trend like the one already observed in transmittance, reaching Δ*T* = 0 ± 1 °C at ε = 125%. During the elastic recovery phase, Δ*T* decreased again to about −5 ± 1 °C for both materials.

Despite the differences in mechanical behavior, with LCE30 showing higher elastic recovery than LCE20, the shielding efficiency was similar for both materials. It is worth noting that achieving the same optical property change, LCE20 required approximately half the mechanical force needed for LCE30, which could lead to a substantial reduction in energy consumption during application as tunable smart windows, making LCE20 more appealing for real applications.

Further experiments were performed to demonstrate the reversibility of the thermal shielding effect during and after removal of the mechanical stretching. The reversibility was tested after the first strain–release cycle used to precondition the sample, so that the initial elongation corresponds to the final value for the sample described in Figure 7 (75% of the initial strain). Samples were then elongated within the region of the elastic behavior, up to 125%, while monitoring the transmittance and the thermal shielding effect. The results are shown in Figure 8 for LCE20, while the same analysis for LCE30 is reported in Figure S9. Throughout these cycles, the integrated transmittance varied between 35% and 80%, closely following the deformation of the specimen without any noticeable hysteresis. Regarding the thermal shielding, the difference between the shielded thermistor and the reference (Δ*T*) varied in the range of 0 ± 1 °C when the LCEs are in transparent mode and −6 ± 1 °C when they are in opaque mode. The tests demonstrated a good reproducibility and reversibility of both the optical and shielding behaviors of LCEs over several switch cycles.

**Figure 8:**
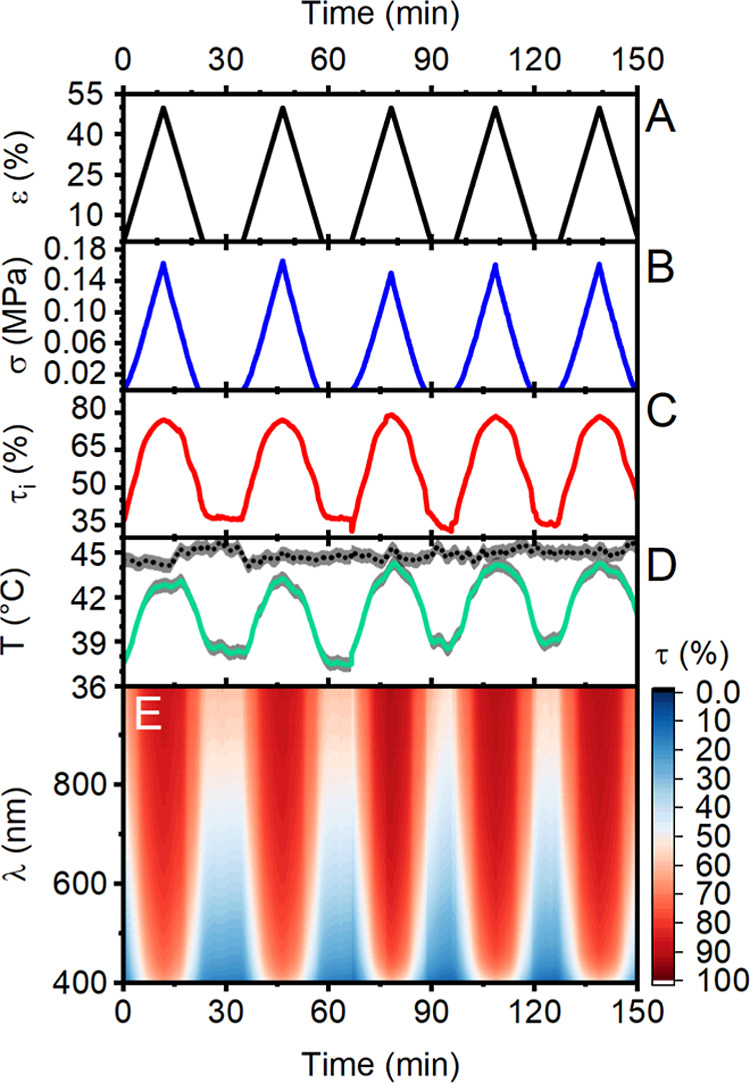
Reversibility of mechano-responsive behavior of LCE20 during different stress–relaxation cycles. (A) Strain over time for different pulling–retracting cycles for the LCE20 sample; (B) stress over time for different cycles; (C) temperature over time measured over different cycles for the shielded thermistor (green curve) and for the reference one (black one); (D) integrated transmittance of the sample from 400 to 1000 nm; and (E) transmittance of the sample over time.

## 4 Conclusions

In this work, polydomain LCEs were synthesized through a base-catalyzed thiol–Michael addition, using a standard and easily scalable technique. Materials with different cross-linker concentrations (LCE20, LCE30, and LCE50) were prepared to investigate the effect of the polymer structure on the mechanical, thermal, and optical properties of the resulting elastomers.

As expected, the materials presented a thermochromic behavior: at a low temperature, in their nematic state, they appeared opaque, while upon heating they reached an optical clearing point beyond which they became transparent. The optical clearing point increased with decreasing cross-linking density, ranging from 25 °C for the highly cross-linked sample (LCE50) to 60 °C for the least cross-linked one (LCE20). The latter being the most suitable for mechano-responsive smart windows that operate in conditions where temperatures should be high (e.g., during summer) without undergoing undesired optical changes. Very interestingly, LCE30 and LCE20 also exhibited a comparable mechano-responsive behavior: starting from an opaque appearance at ε = 0%, the transmittance increased up to 80% for ε = 125%. When the applied load was released, the opacity was partially recovered. During the stress–relaxation cycles, we also demonstrate the shielding capability of the samples: both formulations in the opaque state allowed a temperature decrease of 10 °C between a shaded area and an exposed area. The temperature difference disappeared completely in the transparent state and then decreased again by 5 °C following the elastic recovery.

Finally, to evaluate the repeatability of the optical switching behavior, the materials were subjected to stress–relaxation cycles between 75% and 125% strain, where the elastic recovery was complete. The tests demonstrated an exceptional reproducibility of the adjustable LCE optical responses with a corresponding thermal shielding behavior. Despite its lower elastic properties, LCE20 exhibited mechano-chromic behavior like, and in some cases superior properties, than LCE30 (e.g., higher maximum transmittance), fully matching its thermal shielding performance. These factors, combined with the higher clearing point, make LCE20 candidate of choice for the development of smart mechano-responsive windows or screens with low power consumption. Further studies will be focused on the possible integration of the materials in real buildings, e.g., inside a double-pane glass window, especially focusing on synthesis scale up and long-term stability (e.g., concerning UV resistance, humidity stability, and fatigue life) of the material performances.

## Supplementary Material







## Notes

POM characterizations at different temperatures and during stretching, DSC traces, integrated optical contrast of LCEs at different strain values, thermo-camera images of LCE during stretching, further data on stress–relaxation cycles to demonstrate the reversibility of material deformation and the thermal shielding effect, and a thermo-camera movie of LCE20 during a stress–strain experiment (PDF)
(MP4)
