# Supplementary material for: Polydomain Liquid Crystal Elastomers with Mechanically Switchable Opacity for Thermal Shielding
Source: ACS Polym Au. 2026 Apr 17;6(3):953–62. doi: 10.1021/acspolymersau.6c00032 (PMC13261738; doi:10.1021/acspolymersau.6c00032)
Supplement: Supplementary file 1 [file lg6c00032_si_001.pdf]

Supporting Information

Polydomain Liquid Crystal Elastomers with mechanically-switchable opacity for thermal shielding

Marco Turriani[a,b], Andrea Lanfranchi[c], Diederik S. Wiersma[a,b], Camilla Parmeggiani[b,d], Paola Lova *[c], Daniele Martella.*[b,d]

[a] Dipartimento di Fisica e Astronomia, University of Florence, Via Sansone 1, 50019 Sesto Fiorentino (FI), Italy.
[b] LENS (European Laboratory for Non-Linear Spectroscopy) Via Nello Carrara 1, 50019 Sesto Fiorentino (FI), Italy.
[c] Dipartimento di Chimica e Chimica Industriale, University of Genoa,Via Dodecaneso 31, 16146 Genoa, Italy.
[d] Dipartimento di Chimica "Ugo Schiff", University of Florence, Via della Lastruccia 13, 50019 Sesto Fiorentino (FI), Italy.

E-mail: daniele.martella@unifi.it, paola.lova@unige.it

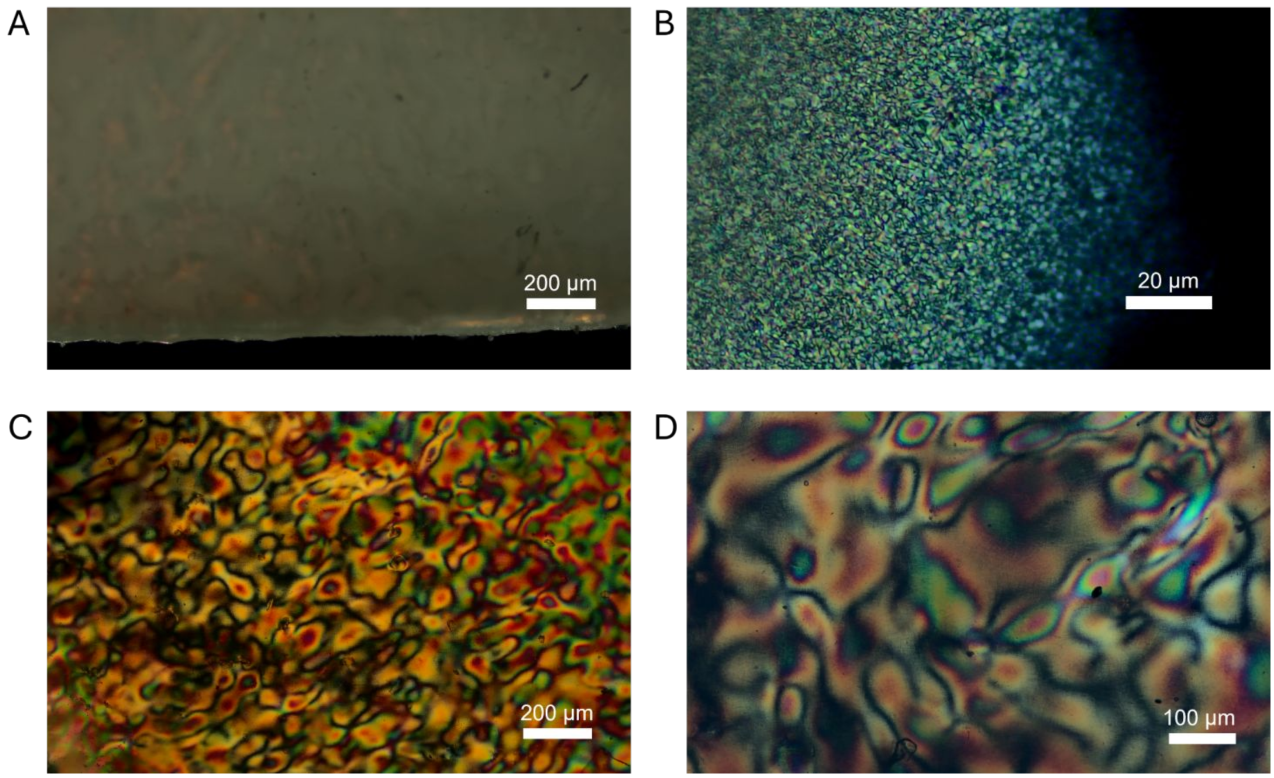

Figure S1. Polarized optical microscope (POM) images of the samples after synthesis. Observing the bulk materials, LCE30 presents a grey/dark image (A) due to the strong light scattering. Only by observing a thin layer of LCE30 (B), we recognize a polydomain structure with domains smaller than 1 µm. A totally different structure is observed for LCE50, where also in the bulk (C and D), very bigger domains appear (> 10 µm). Indeed, increasing the concentration of non-liquid crystalline monomers increases the domain size thus reducing the light scattering.

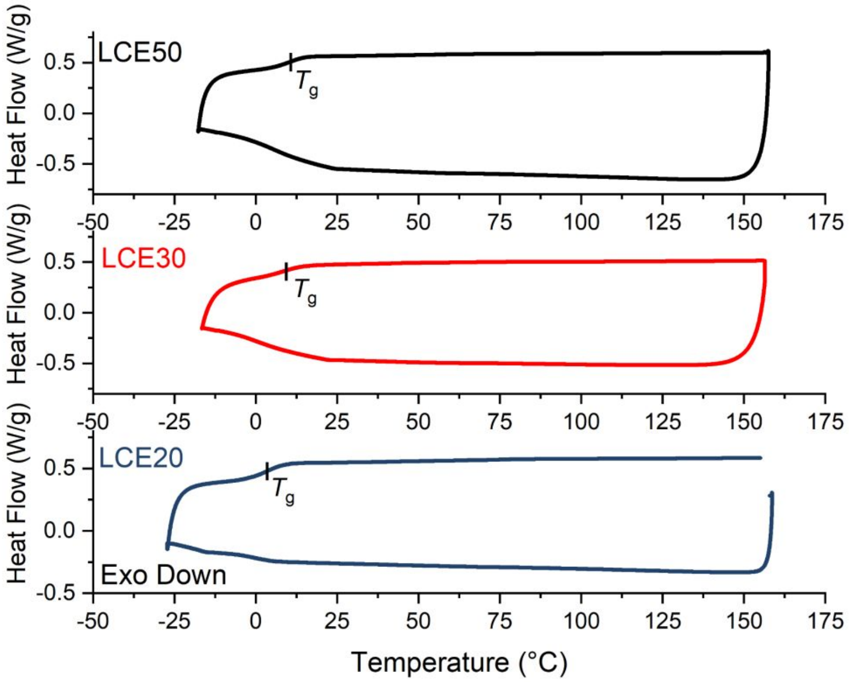

Figure S2. DSC traces of the LCE films. The graph reports the first heating-cooling cycle (20°C/min) for each material, where only the glass transition can be observed. T_g increases with the crosslink density with values of 3, 8 and 11 °C for LCE20, LCE30 and LCE50 respectively.

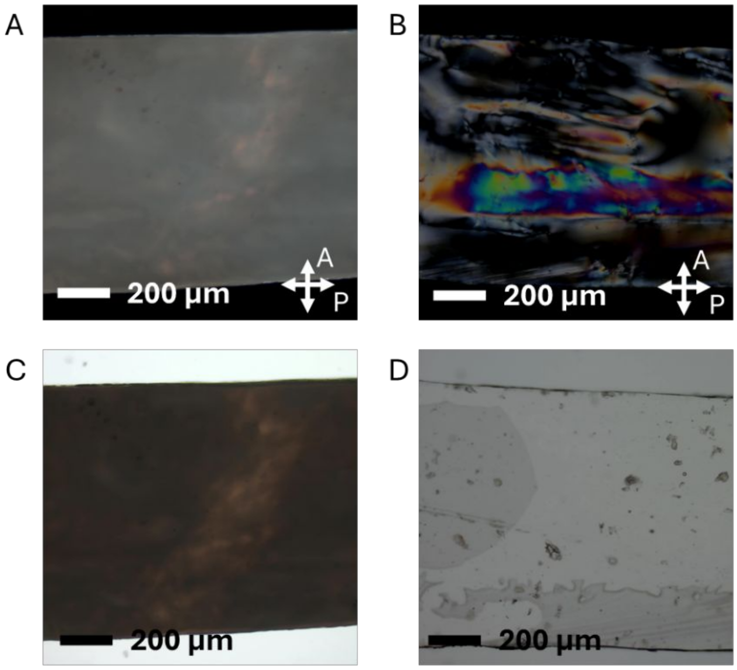

Figure S3. Thermo-responsive behavior of LCE20 under the microscope. A) Polarized optical microscope (POM) image of LCE20 at room temperature. B) POM image of LCE20 at 90 °C. C) Bright field microscope image of LCE20 at room temperature. D) Bright field microscope image of LCE20 at 90 °C.

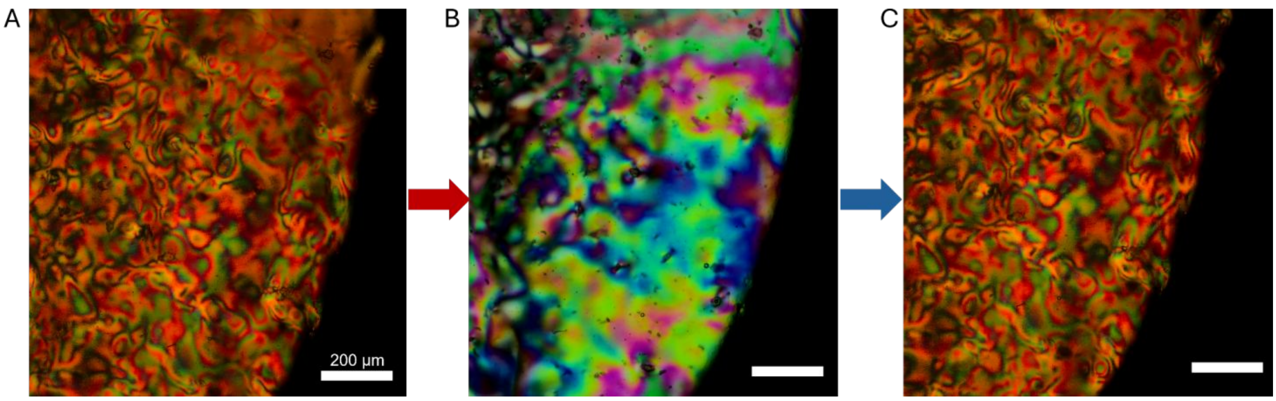

Figure S4. Thermo-responsive behavior of LCE50 under the microscope. POM image of the samples at room temperature (A), at 70 °C (B) and after cooling down at root temperature again (C). Recovery of the initial texture of the polydomain structure was observed after several heating-cooling cycles.

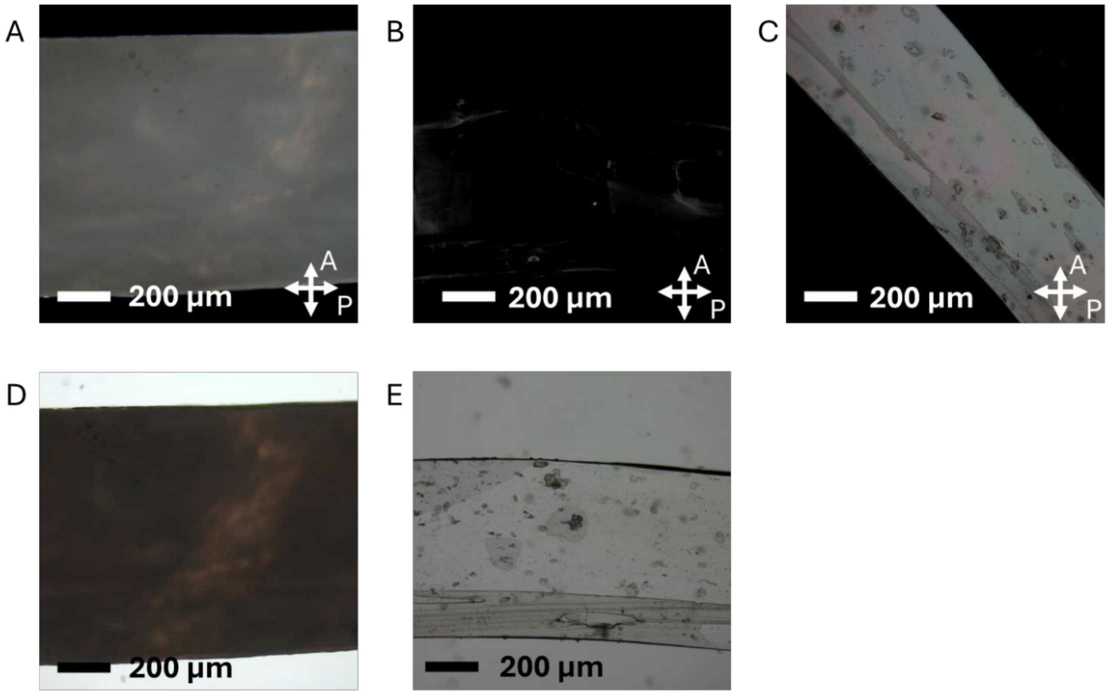

Figure S5. Mechanoresponsivity of LCEs under the microscope. a) Polarized optical microscope (POM) image of LCE20. b) POM image of LCE20, stretched by 110% along the axis parallel to the polarizer. c) POM image of LCE20, stretched by 110% along an axis at 45 ° within the polarizers. d) Bright field microscope image of LCE20. e) Bright field microscope image of LCE20 stretched by110%.

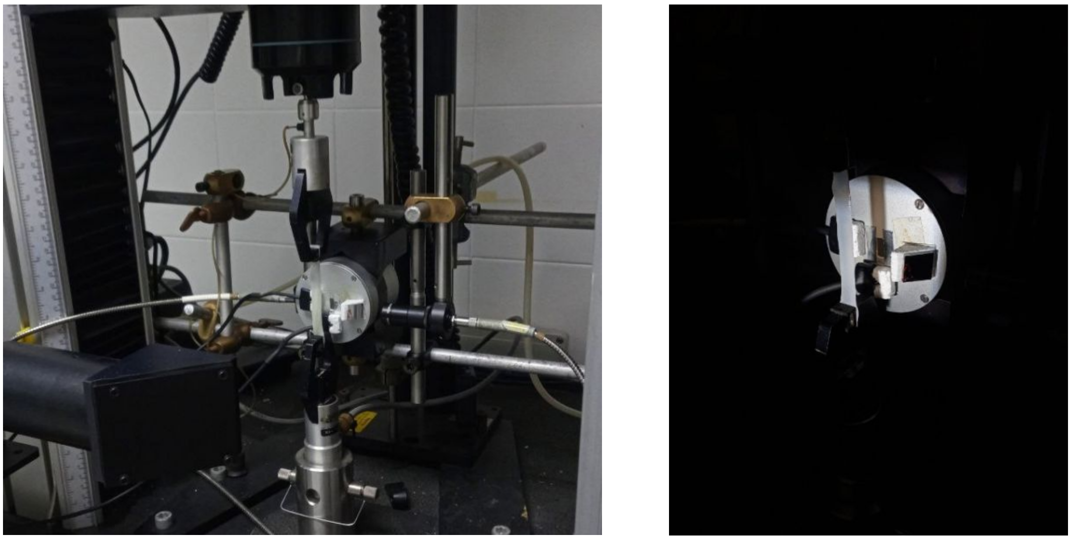

Figure S6. Photo of the set up used for the evaluation of mechano-optical and shielding behaviour.

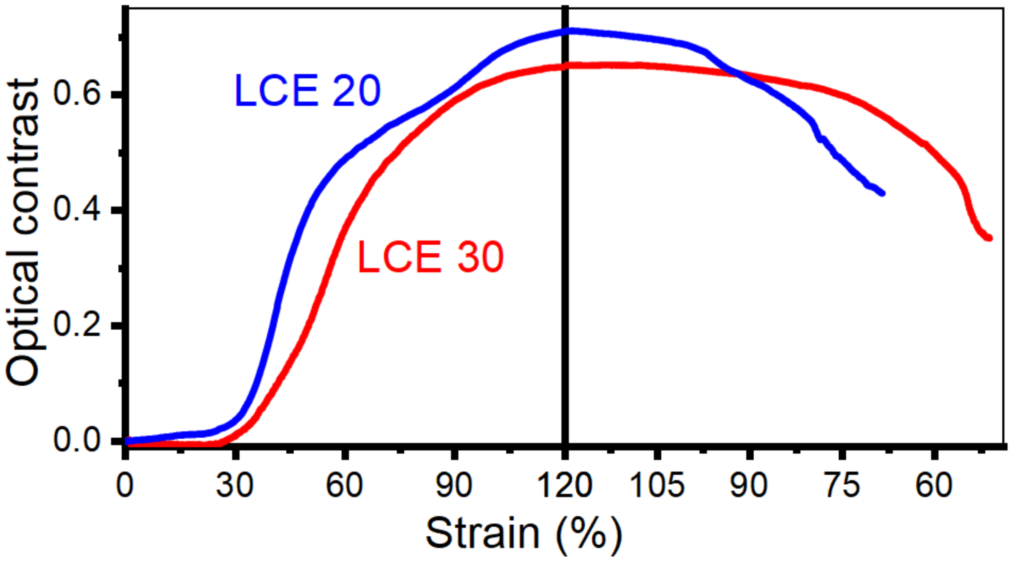

Figure S7. Integrated optical contrast for LCE20 (blue) and LCE30 (red).

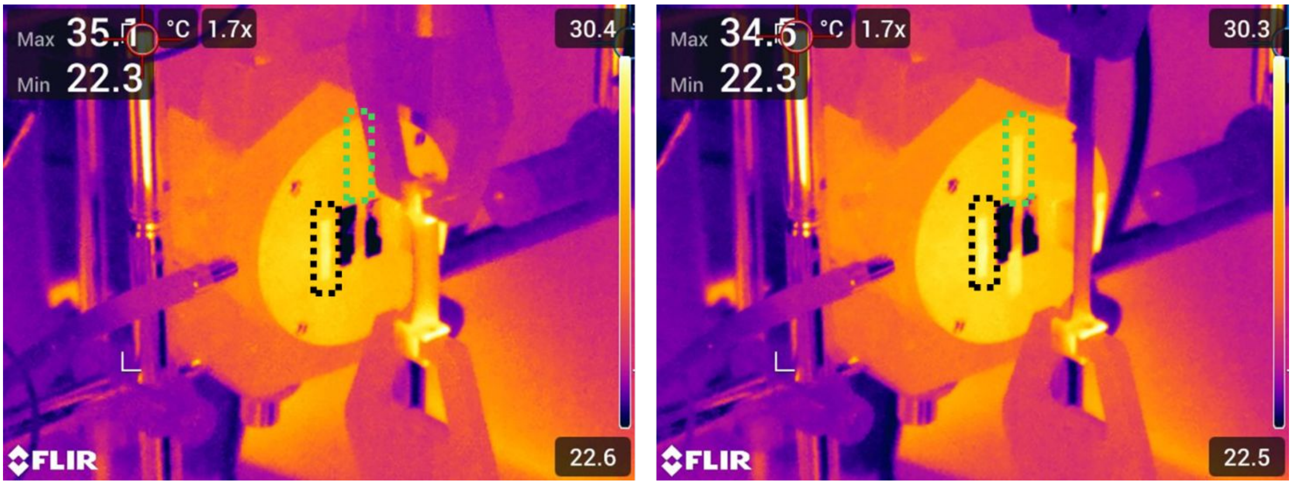

Figure S8. Thermo-camera photos of LCE20 during stretching. On the left LCE20 in the opaque state shield the tape behind it (green rectangle) that has a lower temperature of a reference tape (black rectangle) directly exposed to light. On the right stretched LCE20 in the transparent state doesn't shield anymore the tape behind it that reaches a temperature comparable with the reference one.

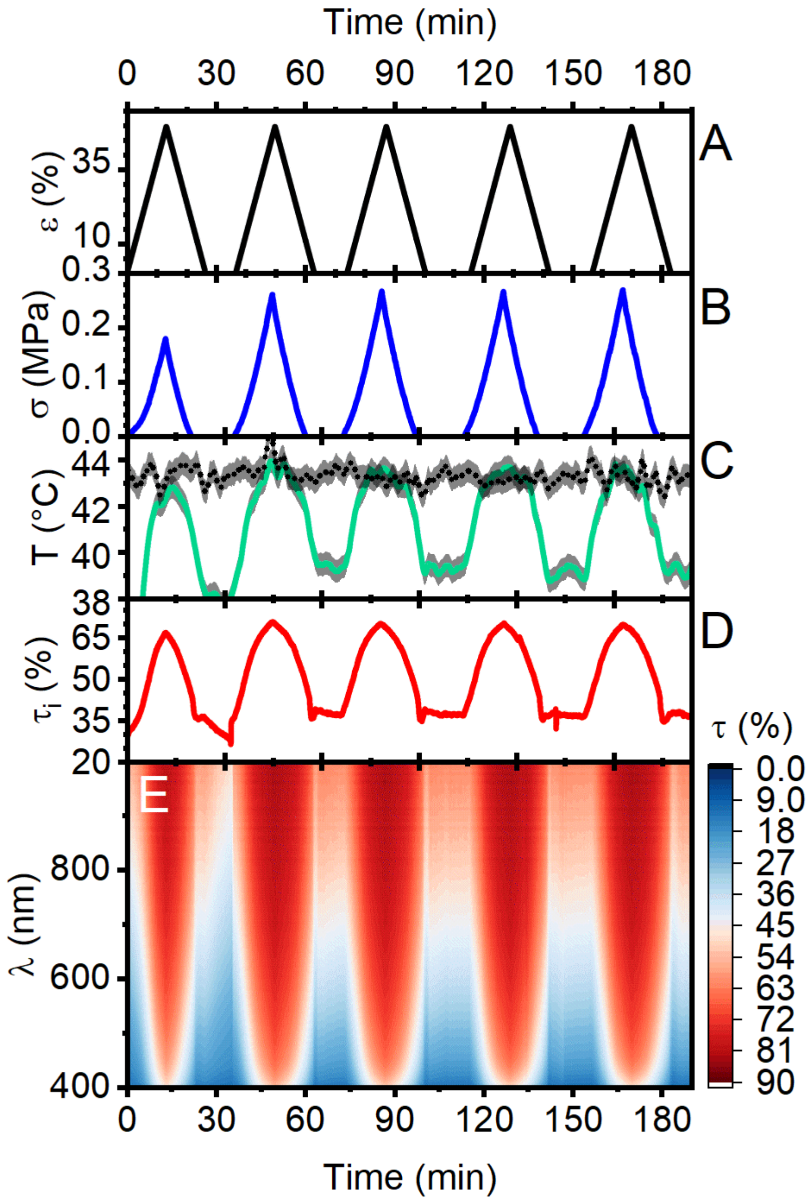

Figure S9. LCE30 stress relaxation cycles. A) Strain over time and different pulling-retracting cycles for LCE30 sample. B) Stress over time over different cycles. C) temperature over time measured over different cycles for the shielded thermistor (green curve) and for the reference one (black one). D) integrated transmittance of the sample from 400 to 1000 nm. E) transmittance of the sample over time.

Movie S1. Thermo-camera movie of LCE20 during a stress-strain experiment. The red arrows highlight the area shaded by the sample, while the white arrow highlights the reference tape.
