# Supplementary figures and images for: Polydomain Liquid Crystal Elastomers with Mechanically Switchable Opacity for Thermal Shielding
Source: ACS Polym Au. 2026 Apr 17;6(3):953–62. doi: 10.1021/acspolymersau.6c00032 (PMC13261738; doi:10.1021/acspolymersau.6c00032)

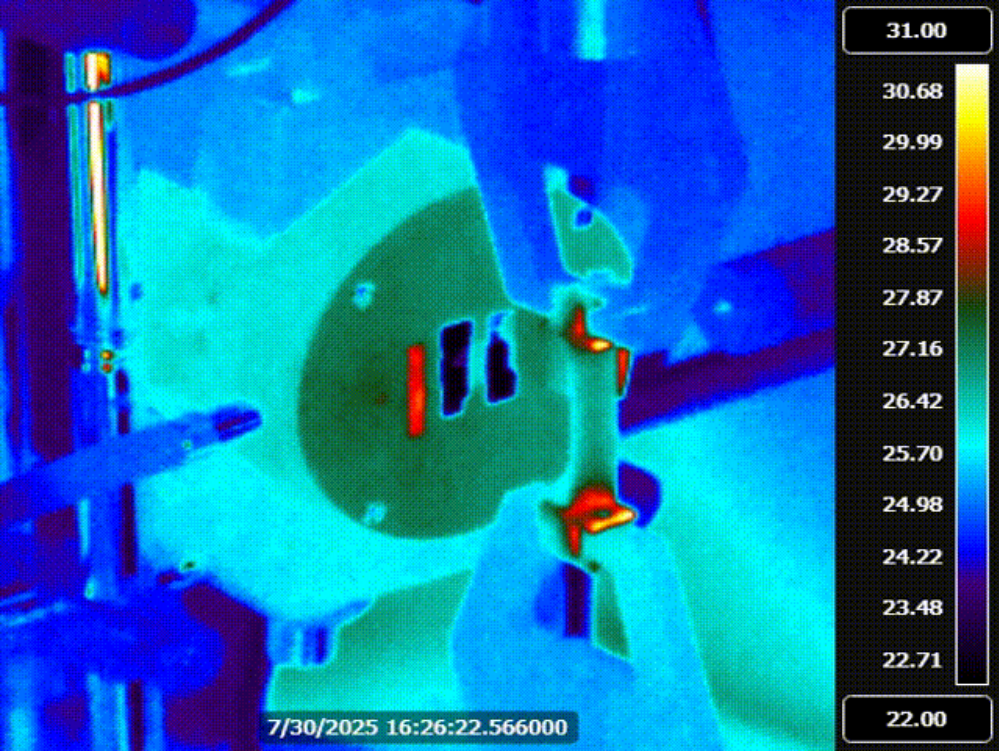

225x169mm (72 x 72 DPI)

Supplement: Supplementary file 2 [file lg6c00032_si_002.pdf]
